# Association between handgrip strength and depressive symptoms in patients undergoing hemodialysis: a cross-sectional study from a single Chinese center

**DOI:** 10.1186/s12888-024-05576-8

**Published:** 2024-03-05

**Authors:** Shuang Zhang, Shu-Xin Liu, Qi-Jun Wu, Zhi-Hong Wang, Hong Liu, Ping Xiao, Yan Lu, Cui Dong, Qing-Mei Meng

**Affiliations:** 1 Department of Nephrology, Dalian Municipal Central Hospital, No.826, Xinan Road, Dalian, Liaoning 116033 P. R. China; 2 Dalian Key Laboratory of Intelligent Blood Purification, Dalian Municipal Central Hospital, Dalian, China; 3 Faculty of Medicine, Dalian University of Technology, Dalian, China; 4 Department of Clinical Epidemiology, Shengjing Hospital of China Medical University, Shenyang, China

**Keywords:** Cross-sectional study, Depressive symptoms, Handgrip strength, Hemodialysis

## Abstract

**Background:**

The relationship between handgrip strength (HGS) and depression in patients undergoing hemodialysis (HD) was unknown. Therefore, we aimed to clarify this association in a cohort of patients.

**Methods:**

HGS was used as a representative indicator of muscle strength and was measured with a handheld dynamometer. Depressive symptoms were assessed with the self-reported Patient Health Questionnaire-9. A multivariable logistic regression model and restricted cubic spline analysis were used to assess the relationship between HGS and depression.

**Results:**

The prevalence of depression in our study was 34% in 568 Chinese patients undergoing HD. Compared with patients in the lowest tertiles of absolute and weighted HGS, patients in the highest tertiles of HGS had an approximately 59% lower [odds ratio (OR) = 0.41, 95% confidence interval (CI) = 0.24–0.68; OR = 0.41, 95%CI = (0.24–0.69)] prevalence of depressive symptoms after multivariate adjustments. Besides, the risk of depression in hemodialysis patients decreased by 33% (OR = 0.67, 95%CI = 0.53–0.85) and 32% (OR = 0.68, 95%CI = 0.54–0.85) for each standard deviation increase in absolute HGS and weighted HGS, respectively. The prevalence of depressive symptoms decreased with both increasing absolute HGS and weighted HGS after multivariate adjustments (*p* for trend < 0.05). Furthermore, a linear dose-response relationship was observed between absolute HGS and weighted HGS and the prevalence of depressive symptoms (*p*_nonlinearity_>0.05).

**Conclusions:**

This study suggests that lower handgrip strength, a simple and modifiable parameter, is associated with a higher prevalence of depression in Chinese patients undergoing HD. Considering that depression is often unrecognized or underdiagnosed in HD patients, lowered muscle strength should be an important indicator and incentive for medical staff to screen for depression.

## Introduction

Depression is a common and serious psychiatric disorder in patients with end-stage renal disease (ESRD) who undergo maintenance hemodialysis (MHD) [1], and has become an important public health problem worldwide. Depression is five times more common in patients with ESRD than in the general population, and as many as 40% of people with ESRD requiring MHD have depression [2]. A multicenter cross-sectional study from northern China has indicated that the prevalence of depression in patients undergoing hemodialysis (HD) is as high as 55% [3]. Patients undergoing MHD who experience depression may have elevated risk of adverse outcomes, such as poorer dialysis treatment outcomes, dietary and medication incompliance, lower quality of life, higher hospitalization rates and higher mortality [4–8]. Therefore, early identification of risk and corresponding management of depression in patients undergoing MHD is essential. According to a previous study, genetics [9], serious illness [10], medication [11], social factors and lifestyle are associated with depression [12]. Moreover, recent studies have focused on assessing the relationship between muscle strength and depression. Several studies have indicated that muscles secrete myokines including irisin and FGF21, which are believed to be associated with the development of depression [13, 14].

Handgrip strength (HGS), an indicator of muscle mass function, is a simple parameter that effectively reflects total muscle strength and is widely used in clinical practice [15]. To date, the relationship between HGS and depression has been extensively researched. Several prospective cohort studies have indicated that decreased HGS is associated with an increased risk of depressive symptoms in the general population [16–18]. Furthermore, a recent meta-analysis including 16 studies has found an association between low muscle strength and intensified depressive symptoms in older populations [19]. However, the populations included in those studies were relatively healthy. In ESRD patients undergoing HD, the frequency of sarcopenia is even greater than among subjects with normal renal function [20], due to uremic-induced anorexia, metabolic disorder, and hormonal derangements inhibit muscle synthesis and accelerate muscle wasting [21]. Besides, dialysis patients tend to have low physical activity, resulting in aggravation of muscle loss [22]. To our knowledge, no previous study has explored the relationship between HGS and depression in patients undergoing MHD. Given the rapid increase in the number of patients undergoing MHD and the high prevalence of depression in patients undergoing MHD, exploring whether HGS is associated with depression should help clinicians and nurses achieve early screening for this factor and provide effective interventions to ameliorate HGS and alleviate depression. Furthermore, such measures could potentially improve quality of life in patients undergoing MHD.

Therefore, we performed a cross-sectional study in a hospital setting to investigate the association between HGS and depression in patients undergoing MHD. We hypothesized that a negative relationship exists between HGS and depression in Chinese patients undergoing MHD. Our results provide new insights into this important issue.

## Methods

### Study design and participants

**Fig. 1:**
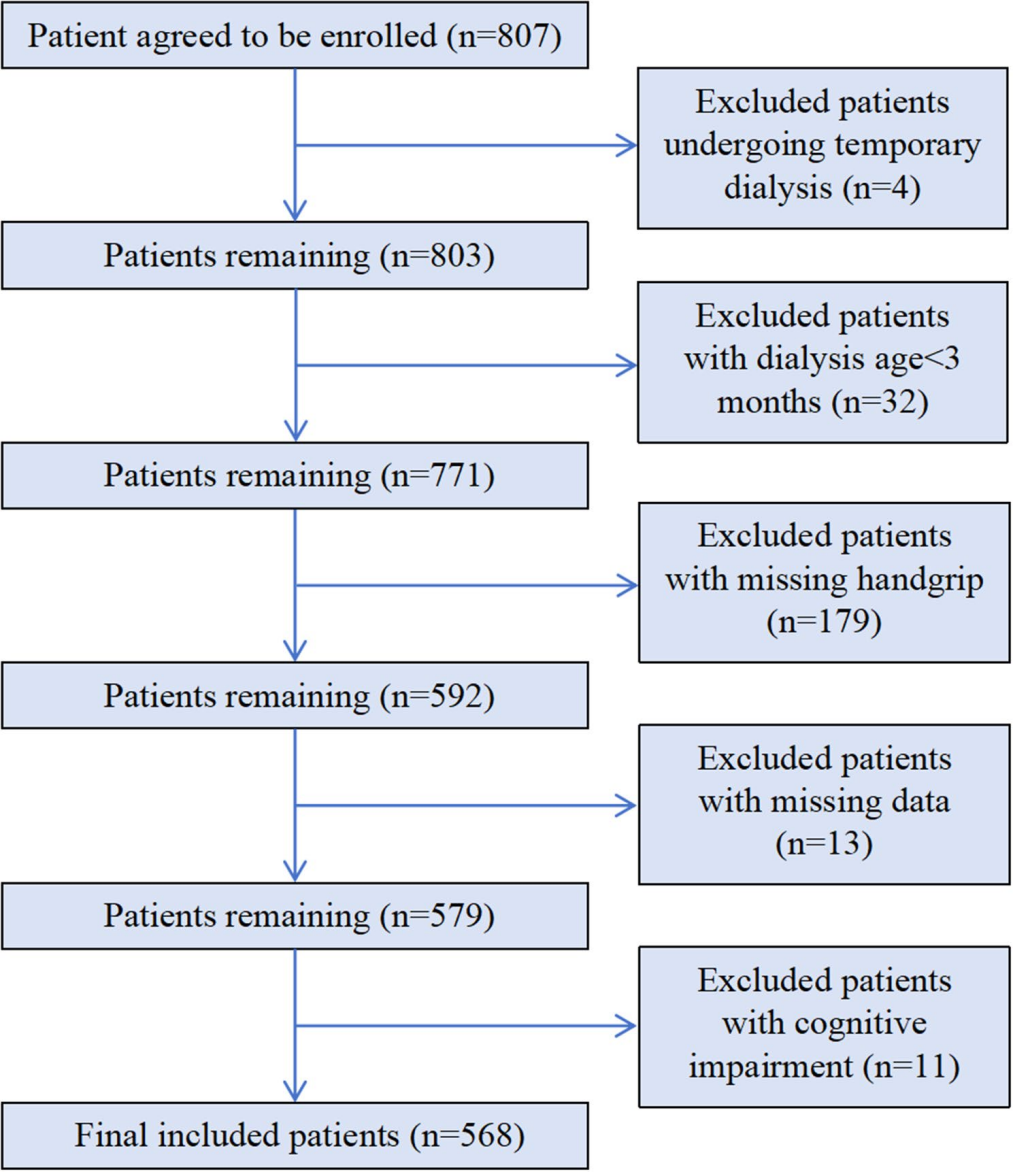
A flow chart indicates patients enrollment

We designed a cross-sectional study, which was performed from December 2021 to January 2022. We enrolled patients undergoing HD who received regular HD treatment at the Dialysis Center of Dalian Municipal Central Hospital, Dalian, China, the largest HD center among the three provinces in northeast China. The eligibility criteria for patients were as follows: age > 18 years; treatment with conventional HD for at least 3 months; normal cognitive function; and ability to complete the Patient Health Questionnaire-9 (PHQ-9). We excluded patients undergoing HD who received temporary dialysis (*n* = 4), patients with < 3 months of dialysis treatment (*n* = 32), patients who did not provide information on HGS (*n* = 179), patients with missing study variables (*n* = 13), and patients with mild impaired cognitive function (*n* = 11). Consequently, 568 patients undergoing MHD were eligible for the final analysis (Fig. 1). All study protocols conformed to the principles of the Declaration of Helsinki and were approved by the Institutional Medical Ethics Committee of Dalian Municipal Central Hospital (protocol number: YN2022-039-05). All study patients provided written informed consent to participate.

### Data collection

Baseline data were obtained from Dalian Municipal Central Hospital electronic medical records or a standardized self-reported questionnaire, including sex, age, duration of dialysis treatment, education level, marital status, annual family income, physical activity, smoking status (at least once per day for > 6 months), alcohol consumption (at least once per day for > 6 months) and concomitant diseases (diabetes mellitus, hypertension and history of cardiovascular disease). Diabetes was defined by a history of diabetes mellitus or use of antihyperglycemic medication. Hypertension was defined by documentation in the medical record or taking antihypertensive drugs. A history of cardiovascular disease was defined by previous angina, transient ischemic attack or cerebrovascular incident, congestive heart failure, myocardial infarction or peripheral arterial disease. After dialysis, a physical examination was performed to measure the patients’ weight. Body height was measured by trained personnel according to a standard protocol. The formula for calculating body mass index (BMI) was as follows: BMI = weight in kilograms divided by the square of the height in meters (kg/m^2^). Physical activity was measured with the International Physical Activity Questionnaire-Short Form, which contains seven questions and has been confirmed to be valid in patients undergoing HD [23]. We used the Montreal Cognitive Assessment (MoCA) to assess the cognitive function of HD patients. The sum of the scale items produces a total MoCA score ranging from 0 to 30, which is positively associated with the global cognitive function [24]. The mild cognitive impairment criteria is based on the Chinese MoCA norms [25]: total MoCA score ≤ 13 for illiterate individuals, ≤ 19 for individuals with 1–6 years of education, and ≤ 24 for individuals with 7 years of education or more.

Blood samples were collected from patients before HD treatment in the middle of the week. The levels of biochemical indicators, including hemoglobin, albumin, calcium, phosphorus, creatinine, C-reactive protein, ferritin and urea nitrogen, were determined according to a standard protocol. Urea clearance was measured with standard methods, and dialysis adequacy was calculated as follows: Kt/V = -ln(R-0.008×t)+(4–3.5×R)×UF/W, where R is the ratio of the serum urea nitrogen concentration after dialysis to the concentration before dialysis, t is the duration of HD in hours, UF is the amount of ultrafiltration in liters during the HD session, and W is the weight after dialysis in kilograms [26].

### Assessment of depressive symptoms

The presence of depression was identified with the PHQ-9, a reliable and valid screening tool for measuring depression severity, over the prior 2 weeks [27]. The PHQ-9 has shown significant screening efficacy in a variety of populations, including Chinese patients in primary care settings [28, 29]. The PHQ-9 score comprises nine questions that are scored from 0 to 3 according to symptom frequency, for a total of 27 points [30]. If PHQ-9 scores are ≥ 5, patients are considered depressed [27].

### Measurement of handgrip strength

Patients were tested by trained evaluators using a hand-held dynamometer (EH101; CAMRY, Guangdong, China) that measured forces between 5.0 and 100.0 kg and had an adjustable grip span. The width of the dynamometer was adjusted to fit each patient optimally. Additionally, the evaluator provided verbal encouragement to elicit maximum power from the patients during the measurement. Patients were asked to stand upright, let their arms fall naturally and not close to the body, and then squeeze the dynamometer with maximum force. This process was repeated twice for each hand. The greatest force was used as the final HGS. In addition, weighted HGS was normalized to body weight to account for the proportion of HGS relative to body weight (HGS [kg]/body weight after dialysis[kg]).

### Statistical analysis

All continuous variables were tested for normality with the Kolmogorov-Smirnov test. Results for continuous variables are expressed as mean ± standard deviation (SD) or median [quartile 1–quartile 3], and comparisons between groups were analyzed with Students t tests for normally distributed data or with Mann-Whitney U tests for nonnormally distributed data. Categorical variables are expressed as numbers and percentages, and differences between groups were analyzed with chi-square tests. Tertiles were categorized on the basis of the distribution of HGS and weighted HGS, and used for further analyzes. The lowest tertile was considered the reference group. Logistic regression models were used to estimate the odds ratios (ORs) and 95% confidence interval (CIs) between HGS and weighted HGS and depressive symptoms for patients undergoing MHD. The crude model was used to calculate the crude OR (95% CI) without any adjustment. The first model adjusted for age (continuous, years), sex (male/female) and BMI (continuous, kg/m^2^). The second model adjusted for time on dialysis (continuous, months) on the basis of model 1. The third model adjusted for physical activity (continuous, metabolic equivalent of task/min/week), smoking status (never/current or former), alcohol intake (never/current or former), education level (junior high school or below, senior high school/secondary specialized school/junior college/university or above), marital status (unmarried/married/divorced/widowed) and co-morbidities (yes or no), on the basis of model 2. Logistic regression analysis was used to determine linear trends by allocation of the median HGS and weighted HGS for each tertile as a continuous variable. The interaction test was performed by simultaneous addition of corresponding multiplication terms to the model. Subgroup analyses based on sex, age, BMI and duration of dialysis treatment were also performed. The dose-response relationship between absolute HGS and weighted HGS and depression syndrome was assessed with a restricted cubic spline (RCS) regression model with four nodes at the 5th, 35th, 65th and 95th percentiles, adjusting for the aforementioned confounders [31]. All analyses were performed in SAS (version 9.4; SAS Institute Inc., Cary, NC, USA). The level of statistical significance was set at *p* < 0.05 and was based on a two-sided test.

## Results

### Baseline characteristics of the patients

The baseline detailed characteristics are presented in Table 1. The final study included 568 patients undergoing MHD, based on our inclusion and exclusion criteria, and the median age was 59 years (Interquartile range, IQR = 18). Overall, the prevalence of depression was 34%, and the median absolute HGS and weighted HGS were 23.30 kg (IQR = 10) and 0.34 kg/kg (IQR = 0.16), respectively. In this study, the number of males (63.2%) was higher than that of females (36.8%). Compared with patients without depression, patients with depression tended to have lower physical activity, annual family income and grip strength, and higher rates of being divorced or widowed (all *p* < 0.05).

**Table 1:** Baseline characteristics of study patients according to the states of depressive symptoms

Characteristics	All patients (568)	Depressive symptoms		*p*-value
		Non depression group (*n* = 375)	Depression group (*n* = 193)	
**Demographics**				
Age (years)	59 (49–67)	58 (49–67)	60 (47–67)	0.773
Male (n, %)	359 (63.20)	246 (65.60)	113 (58.55)	0.099
Time on dialysis (months)	50 (22–104)	48 (21–105)	56 (25–103)	0.386
BMI (kg/m^2^)	23.91 (21.48–26.35)	23.88 (21.64–26.51)	23.96 (21.42–26.21)	0.579
Physical activity (MET/min/week)	489 (0−1386)	594 (0−1386)	264 (0−897)	< 0.001
Primary kidney disease (n, %)				0.811
Diabetic nephropathy	179 (31.51)	113 (30.13)	66 (34.20)	
Glomerulonephritis	210 (36.97)	142 (37.87)	68 (35.23)	
Hypertensive benign renal arteriosclerosis	70 (12.32)	49 (13.07)	21 (10.88)	
Polycystic kidney	37 (6.51)	25 (6.67)	12 (6.22)	
Others	72 (12.68)	46 (12.27)	26 (13.47)	
Educational level (n, %)				0.057
Junior secondary or below	259 (45.60)	177 (47.20)	82 (42.49)	
Senior high school/technical secondary school	173 (30.46)	102 (27.20)	71 (36.79)	
Junior college/university or above	136 (23.94)	96 (25.60)	40 (20.73)	
Marital status (n, %)				0.002
Unmarried	56 (9.86)	29 (7.73)	27 (13.99)	
Married	436 (76.76)	306 (81.60)	130 (67.36)	
Divorced	39 (6.87)	22 (5.87)	17 (8.81)	
Widowed	37 (6.51)	18 (4.80)	19 (9.84)	
Annual family income (RMB thousand yuan), (n, %)				0.039
< 50	169 (29.75)	101 (26.93)	68 (35.23)	
50 to < 100	259 (45.60)	171 (45.60)	88 (45.60)	
≥ 100	140 (24.65)	103 (27.47)	37 (19.17)	
Current or former smoker, (n, %)	259 (45.60)	174 (46.40)	85 (44.04)	0.593
Current or former drinker, (n, %)	211 (37.15)	140 (37.33)	71 (36.79)	0.899
Co-morbid conditions, (n, %)				0.084
Diabetes	225 (39.61)	152 (40.53)	73 (37.82)	
Hypertension	476 (83.80)	321 (85.60)	155 (80.31)	
CVD	332 (58.45)	228 (60.80)	104 (53.89)	
**Laboratory parameters**				
Hemoglobin (g/L)	114 (106–122)	113 (105–122)	115 (107–123)	0.506
Albumin (g/L)	41.70 ± 2.96	41.81 ± 2.85	41.47 ± 3.16	0.194
Calcium (mmol/L)	2.14 (2.03–2.25)	2.13 (2.03–2.25)	2.14 (2.03–2.25)	0.865
Phosphorus (mmol/L)	2.04 (1.69–2.46)	2.05 (1.69–2.45)	2.03 (1.68–2.47)	0.828
Creatinine (µmol/L)	925 (776–1087)	929 (784–1099)	898 (768–1068)	0.098
C-reactive protein (mg/L)	3.30 (3.30–6.84)	3.30 (3.30–6.51)	3.30 (3.30–7.25)	0.460
Ferritin (mg/L)	96.28 (44.84−176.78)	94.66 (43.09–170.90)	102.67 (50.53–184.70)	0.203
Urea nitrogen (mmol/L)	28.48 (24.65–32.89)	28.88 (24.59–33.80)	27.99 (24.67–32.11)	0.072
spKt/V_urea_	1.32 (1.17–1.49)	1.32 (1.16–1.48)	1.32 (1.18–1.50)	0.540
**Handgrip strength**				
Absolute handgrip strength (kg)	23.30 (18.70–28.70)	24.40 (19.50–30.40)	20.80 (17.80–26.00)	< 0.001
Weighted handgrip strength (kg/kg)	0.34 (0.27–0.43)	0.35 (0.28–0.44)	0.31 (0.26–0.40)	0.001

Data presented as mean ± SD (standard deviation) or median (quartile1- quartile3) (continuous variables) or number (%) (categorical variables);

Abbreviations: MET, metabolic equivalents of task; BMI: Body mass index; spKt/V_urea_, Single-pool Kt/Vurea;

*p*-values were determined with Students t tests or the Mann-Whitney U tests for continuous variables, and the chi-square tests for categorical variables

### HGS and depressive symptoms

The associations between absolute HGS and the prevalence of depressive symptoms are presented in Table 2. Absolute HGS was negatively associated with the prevalence of depressive symptoms before and after adjustment for confounding factors (all *p*_trend_ < 0.05). The multivariable adjusted ORs (95% CIs) for prevalence of depressive symptoms across tertiles of absolute HGS were 1.00 (reference), 0.54 (0.34–0.85) and 0.41 (0.24–0.68). The OR (95% CI) for depressive symptom prevalence per 1 SD increase in absolute HGS was 0.67 (0.53–0.85).

**Table 2:** Association between absolute handgrip strength (kg) and depressive symptoms in hemodialysis patients

	Tertiles of absolute handgrip strength			p for trend^a^	per SD increase
	T1	T2	T3		
Absolute handgrip strength (kg)	16.067 (15.568–16.566)^b^	23.237 (22.967–23.507)	33.995 (33.048–34.942)		
No. of patients	187	190	191		
Crude model	Reference (1.00)	0.58 (0.38–0.88)^c^	0.41 (0.27–0.63)	< 0.001	0.69 (0.57–0.83)
Adjusted model 1^d^	Reference (1.00)	0.56 (0.36–0.86)	0.38 (0.23–0.63)	< 0.001	0.65 (0.52–0.82)
Adjusted model 2^e^	Reference (1.00)	0.55 (0.35–0.85)	0.37 (0.23–0.62)	< 0.001	0.65 (0.51–0.81)
Adjusted model 3^f^	Reference (1.00)	0.54 (0.34–0.85)	0.41 (0.24–0.68)	0.001	0.67 (0.53–0.85)

^a^*p*-value for linear trend calculated from category median values;

^b^Mean (95% confidence interval) (all such values);

^c^Odds ratio (95% confidence interval) (all such values);

^d^ Adjusted for age, sex, and body mass index;

^e^ Additionally adjusted for time on dialysis based on Model 1;

^f^ Additionally adjusted for physical activity, educational level, household income, smoking status, drinking status, marital status and comorbidities based on Model 2

The associations between weighted HGS and the prevalence of depressive symptoms are presented in Table 3. Similarly, weighted HGS was also negatively associated with the prevalence of depressive symptoms before and after adjustment for confounding factors (all *p*_trend_ < 0.05). The multivariable adjusted ORs (95% CIs) for depressive symptom prevalence across tertiles of weighted HGS were 1.00 (reference), 0.67 (0.42–1.07) and 0.41(0.24–0.69). The OR (95% CI) for depressive symptom prevalence per 1 SD increase in weighted HGS was 0.68 (0.54–0.85).

**Table 3:** Association between weighted handgrip strength (handgrip strength/weight, kg/kg) and depressive symptoms in hemodialysis patients.

	Tertiles of weighted handgrip strength			p for trend^a^	per SD increase
	T1	T2	T3		
Weighted handgrip strength (kg/kg)	0.233 (0.226–0.240)^b^	0.341 (0.337–0.345)	0.487 (0.476–0.498)		
No. of patients	189	189	190		
Crude model	Reference (1.00)	0.76 (0.50–1.16)^c^	0.50 (0.32–0.76)	0.002	0.74 (0.62–0.89)
Adjusted model 1^d^	Reference (1.00)	0.67 (0.43–1.04)	0.39 (0.23–0.64)	< 0.001	0.67 (0.53–0.82)
Adjusted model 2^e^	Reference (1.00)	0.67 (0.43–1.04)	0.38 (0.23–0.63)	< 0.001	0.66 (0.53–0.82)
Adjusted model 3^f^	Reference (1.00)	0.67 (0.42–1.07)	0.41 (0.24–0.69)	< 0.001	0.68 (0.54–0.85)

^a^*p*-value for linear trend calculated from category median values;

^b^Mean (95% confidence interval) (all such values);

^c^Odds ratio (95% confidence interval) (all such values);

^d^ Adjusted for age, sex, and body mass index;

^e^ Additionally adjusted for time on dialysis based on Model 1;

^f^ Additionally adjusted for physical activity, educational level, household income, smoking status, drinking status, marital status and comorbidities based on Model 2

### Non-linear relationship analyses

**Fig. 2:**
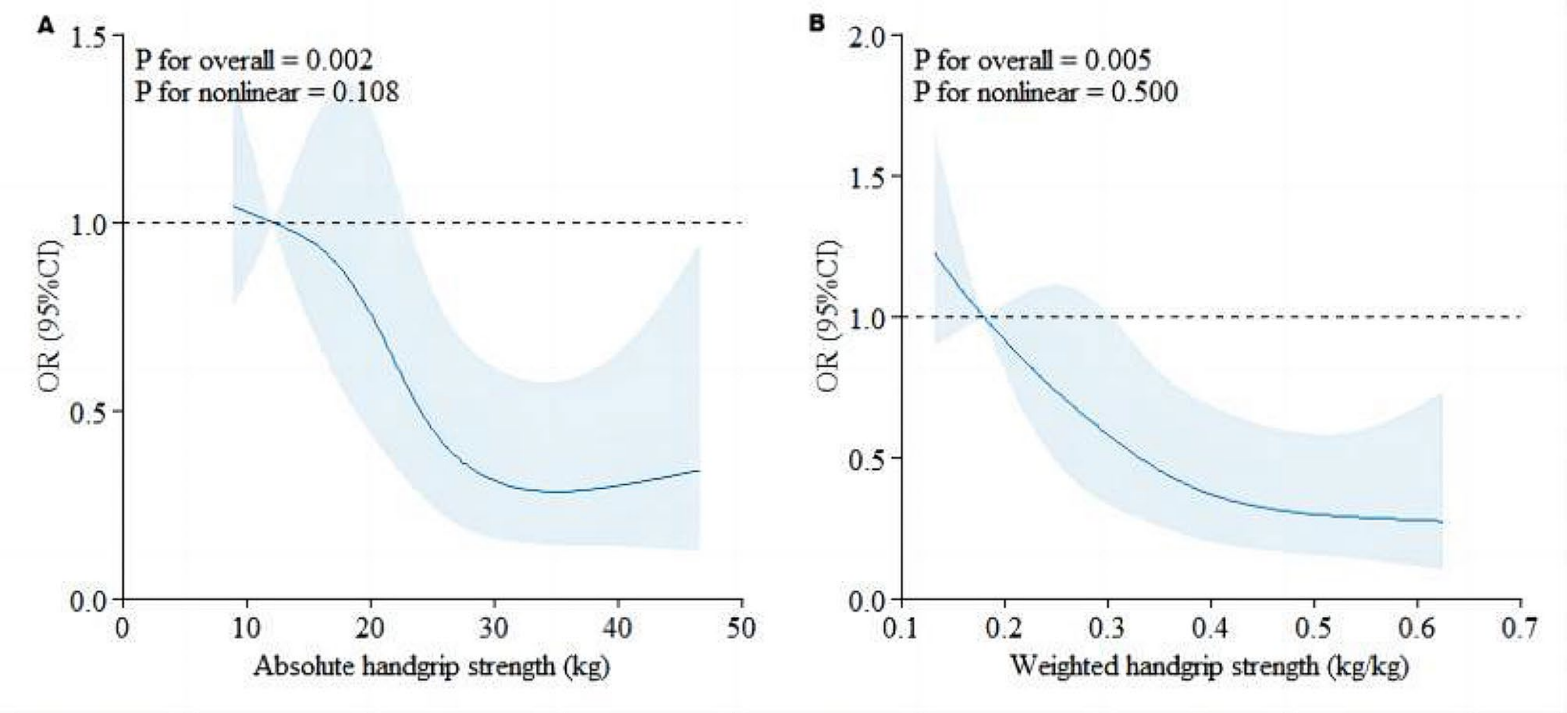
The dose-response curve of the relationship between absolute handgrip strength (**A**), weighted handgrip strength (**B**) and depressive symptoms. The blue line and shaded area represent the estimated odds ratio and 95% confidence interval

An RCS regression model was applied to determine whether a nonlinear relationship existed between HGS and depression. Both absolute HGS and weighted HGS were negatively associated with the prevalence of depressive syndromes; therefore, the likelihood of depressive syndromes decreased with increasing HGS (all *p*_*nonlinear*_ > 0.05) **(**Fig. 2**)**.

### Subgroup analysis of the association between HGS and depressive symptoms

**Fig. 3:**
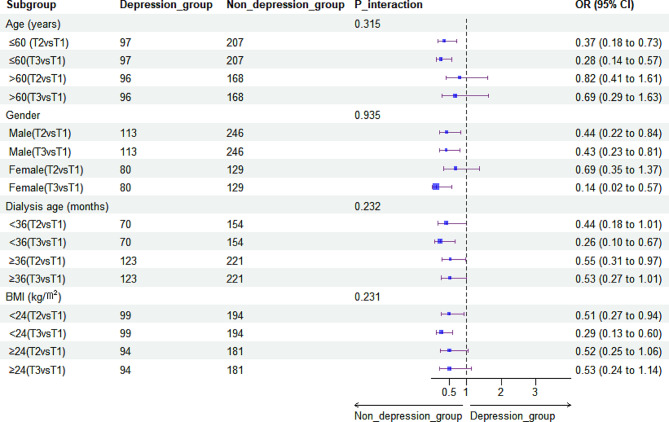
Stratified analyses for the adjusted odds ratio (OR) and 95% confidence interval (CI) for the association between absolute handgrip strength and the prevalence of depressive symptoms

**Fig. 4:**
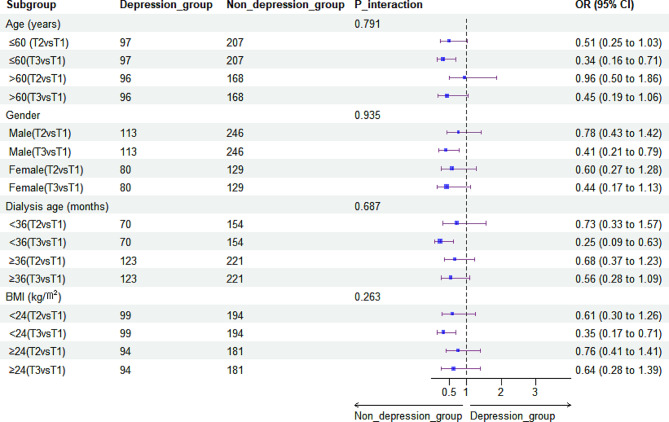
Stratified analyses for the adjusted odds ratio (OR) and 95% confidence interval (CI) for the association between weighted handgrip strength and the prevalence of depressive symptoms

Subgroup analysis indicated that the association between HGS and depressive syndromes in patients undergoing HD was almost unchanged among strata, thus suggesting that this association is reliable and stable (all *p* > 0.05) **(**Figs. 3 and 4**)**.

## Discussion

The present study showed that patients undergoing MHD with low HGS had a higher prevalence of depression after controlling for potential confounders. Our results support the hypothesis that HGS is inversely correlated with depressive symptoms in patients undergoing MHD. In addition, a linear relationship appears to exist between HGS and depression.

Several epidemiological studies have reported the associations between HGS and depressive symptoms in specific populations, such as general populations, rural Chinese populations and cancer survivors. However, these results remain controversial. For example, Bertonia et al. conducted a cohort study using data from 12 European countries participating in the Survey of Health, Aging and Retirement in Europe, and observed no significant association between dynapenia (defined as HGS < 20 kg for women and 30 kg for men) at baseline or the occurrence of dynapenia between baseline and the 2-year follow-up, and the occurrence of depressive symptoms at the 4-year follow-up [32]. However, a prospective cohort study conducted by Cabanas et al., using data from the UK Biobank and including 162,167 participants, indicated that HGS was inversely associated with the occurrence of depression [17]. Another cohort study in 13,208 participants in the China Health and Retirement Longitudinal Study conducted by Lian and colleagues has found that participants in the lowest quartile of HGS had an approximately 36% greater risk of depressive symptoms than participants in the highest quartile [18]. In addition, Zhao et al. have observed the same association between HGS and depression in a prospective cohort study of 8470 participants living in 450 urban communities and rural villages in 28 provinces of China [33]. Similarly, a negative association between HGS and the prevalence of depressive symptoms has been found in 876 cancer survivors (OR = 0.95, 95%CI: 0.92–0.99) [34]. In agreement with previous studies, we observed that a lower HGS was associated with higher prevalence of depressive symptoms in a cohort of patients undergoing MHD. Although the absolute value of HGS has been used in previous studies examining the association between muscle strength and depressive symptoms, according to a previous study [33], the links of muscle strength with both physical function and chronic health are mediated by the proportion of strength relative to body mass; therefore, a normalized HGS value by weight might be suitable for exploring the associations between muscle strength and health outcomes. Although BMI was adjusted for in several previous epidemiological studies, the residual effect of weight remained. Therefore, in the present study, we further investigated the associations between weighted HGS and the prevalence of depressive symptoms. Consequently, we observed the same association between weighted HGS or absolute HGS and the prevalence of depressive symptoms. In fact, patients undergoing MHD usually have poorer disease status and numerous complications. Our study showed that patients undergoing MHD with low HGS were at elevated risk of depression, thus highlighting a need for medical staff to pay greater attention to these patients. Early screening for HGS and encouraging patients to engage in effective physical activity may help decrease the likelihood of depression.

Muscular weakness, muscle wasting and exercise intolerance are components of the uremic myopathy [35], which is a term used in common deficiency of muscle function in patients with ESRD [36, 37]. The exact etiology of uremic myopathy remains uncertain, but it is likely that it has a multifactorial origin. A previous study have indicated that hyperkalemia and acidosis, which are often seen in ESRD patients [38], are two such likely contributing factors, which could account for the functional abnormalities without generating structural changes [39, 40]. Several laboratory and clinical evidence indicates that metabolic acidosis leads to loss of muscle mass via the increased protein breakdown and possibly decreased protein synthesis [41, 42]. Two major pathways have been identified to explain the higher protein breakdown associated with metabolic acidosis-increased activities of both the adenosine triphosphate-dependent ubiquitin-proteasome system and of the branched-chain ketoacid dehydrogenase [43]. According to a previous study conducted by Ikizler et al. in dialyzed patients, the HD procedure per sestimulates protein degradation and reduced protein synthesis with the effect persisting for two hours following dialysis [44].

The mechanisms underlying the associations between low HGS and depression are not clearly understood but appear to be complicated and multifactorial. Previous studies have shown that skeletal muscle is an endocrine organ that secretes myokines. Muscle contraction leads to changes in the environment of the skeletal muscle and thus affects the physiological state of myokines [45]. Myokines act as “training factors” that improve brain function. Several previous studies have shown that myokines such as brain-derived neurotrophic factor [46], irisin [14] and FGF21 [47] act on the nervous system through gene regulation and various endocrine signaling pathways. In addition, improving muscle function may decrease inflammation [48] and oxidative free radicals [49], thereby diminishing the risk of depressive symptoms [50]. Finally, low HGS influences depression through psychosocial mechanisms. As we observed, patients undergoing MHD with depression typically experienced a decline in physical activity, thereby decreasing the likelihood of social contact with others and increasing the likelihood of developing depression through a vicious cycle.

This study has several strengths. To our knowledge, this is the first study to examine the association between HGS and depressive symptoms in a cohort of patients undergoing MHD. Understanding this association is essential for the prevention and treatment of depression in patients undergoing HD. Second, the analyses provided comprehensive results regarding the associations between weighted/absolute HGS and the prevalence of depressive symptoms. Third, an RCS regression model was used to estimate the detailed dose-response relationship between absolute HGS and weighted HGS and depression, thus providing practical suggestions. Fourth, we used standardized instruments to measure muscle strength, which is easily measured and can be standardized across different groups and studies. Finally, our results suggested that increasing HGS through lifestyle modification is a useful strategy for preventing depression in patients undergoing MHD. However, this study has several limitations. First, because this was a cross-sectional study, it has limited ability to indicate a complete causal relationship. More prospective cohort studies are needed for further investigation. Second, because of the limitations in the original data, although we adjusted for some potential confounders, many unknown factors or residual confounding might potentially affect the relationship between HGS and depressive symptoms. Third, depressive symptoms were assessed with self-reported questionnaires rather than diagnostic psychiatric interviews, thus potentially resulting in bias. Finally, the patients were recruited were from a single center; therefore, the conclusions may not be representative of all patients undergoing HD. In addition, the relatively small sample size might have caused some analyses to be insufficiently informative.

In conclusion, our study showed that higher levels of HGS were associated with a lower risk of depressive symptoms in patients undergoing MHD, thus suggesting that improving HGS may have potentially beneficial effects on depressive symptoms. Furthermore, the association between HGS and depressive symptoms in patients undergoing MHD should be investigated in a large cohort study to clarify this finding.

## Data Availability

The datasets used and/or analyzed during the current study are available from the corresponding author upon reasonable request.

## Abbreviations

BMI: Body mass index
CI: Confidence interval
ESRD: End-stage renal disease
HD: Hemodialysis
HGS: Handgrip strength
IQR: Interquartile range
MHD: Maintenance hemodialysis
OR: Odds ratio
PHQ-9: Patient Health Questionnaire-9
RCS: Restricted cubic spline
SD: Standard deviation
